# 

**Published:** 1900-05

**Authors:** 


### CONTENTS.

ORIGINAL COMMUNICATIONS. PAGE

A Suspension Crown. By Horatio C. Meriam, Salem, Mass. . . . . . . . . 293
Hæmophilia. By Charles A. Porter, M.D., Boston, Mass. . . . . . . . . 295
A Fatal Case of Hæmophilia. By Thomas Fillebrown, M.D., D.M.D. . . . 302
Consequences of the Extraction of Permanent Teeth. By E. A. Bogue, M.D., New York, N. Y. . . . . . . . . . . . . . . . . . . . . . . . . 305

ABSTRACTS AND TRANSLATIONS.

On the Rôle of Systemic Hyperacidity, and of Sulphocyanides in the Saliva, in Chemical Abrasion of the Teeth. (*Continued.*) By M. Michaels . . . . . 316

REPORTS OF SOCIETY MEETINGS.

The New York Institute of Stomatology . . . . . . . . . . . . . . . 329
American Academy of Dental Science . . . . . . . . . . . . . . . . 338
Academy of Stomatology . . . . . . . . . . . . . . . . . . . . . 349
Massachusetts Dental Society . . . . . . . . . . . . . . . . . . . 358

EDITORIAL.

Is Dentistry a Charitable Profession? . . . . . . . . . . . . . . . 359
National Association of Dental Faculties . . . . . . . . . . . . . . 363

BIBLIOGRAPHY . . . . . . . . . . . . . . . . . . . . . . . . . . 364

OBITUARY.

Resolutions of Respect to Dr. W. D. Tenison . . . . . . . . . . . . 366

CURRENT NEWS.

National Dental Association . . . . . . . . . . . . . . . . . . . 366
American Medical Association, Section on Stomatology . . . . . . . . 367
National Association of Dental Faculties . . . . . . . . . . . . . . 368
New York State Dental Society . . . . . . . . . . . . . . . . . . 368
National Association of Dental Examiners . . . . . . . . . . . . . 369
Kentucky State Dental Association . . . . . . . . . . . . . . . . 369
Dental Commissioners of Connecticut . . . . . . . . . . . . . . . 370
Pennsylvania State Dental Society . . . . . . . . . . . . . . . . 371
Illinois State Dental Society . . . . . . . . . . . . . . . . . . 371
Missouri State Dental Association . . . . . . . . . . . . . . . . 372
Chicago Dental Society . . . . . . . . . . . . . . . . . . . . . 372

